# Juvenile social defeat stress exposure favors in later onset of irritable bowel syndrome-like symptoms in male mice

**DOI:** 10.1038/s41598-021-95916-5

**Published:** 2021-08-11

**Authors:** Kenjiro Matsumoto, Kana Takata, Daisuke Yamada, Haruki Usuda, Koichiro Wada, Maaya Tada, Yoshiyuki Mishima, Shunji Ishihara, Syunji Horie, Akiyoshi Saitoh, Shinichi Kato

**Affiliations:** 1 Division of Pathological Sciences, Department of Pharmacology and Experimental Therapeutics, Kyoto Pharmaceutical University, Misasagi 5, Yamashina, Kyoto, 607-8414 Japan; 2 Laboratory of Pharmacology, Faculty of Pharmaceutical Sciences, Tokyo University of Science, Chiba, Japan; 3 Department of Pharmacology, Shimane University Faculty of Medicine Graduate School of Medicine, Shimane, Japan; 4 Department of Internal Medicine II, Shimane University School of Medicine, Shimane, Japan; 5 Laboratory of Pharmacology, Josai International University, Chiba, Japan

**Keywords:** Neuroscience, Gastroenterology

## Abstract

Irritable bowel syndrome (IBS) is the most common functional gastrointestinal disorder. Traumatic stress during adolescence increases the risk of IBS in adults. The aim of this study was to characterize the juvenile social defeat stress (SDS)-associated IBS model in mice. Juvenile mice were exposed to an aggressor mouse for 10 min once daily for 10 consecutive days. Behavioral tests, visceral sensitivity, immune responses, and fecal bacteria in the colon were evaluated in 5 weeks after SDS exposure. Social avoidance, anxiety- and depression-like behavior, and visceral hypersensitivity were observed. Juvenile SDS exposure significantly increased the number of 5-HT-containing cells and calcitonin gene-related peptide-positive neurons in the colon. The gut microbiota was largely similar between the control and juvenile SDS groups. The alterations in fecal pellet output, bead expulsion time, plasma corticosterone concentration, and colonic 5-HT content in response to restraint stress were exacerbated in the juvenile SDS group compared with the control group. The combination of juvenile SDS and restraint stress increased the noradrenaline metabolite 3-Methoxy-4-hydroxyphenylglycol (MHPG) content and MHPG/noradrenaline ratio in the amygdala when compared with restraint stress in control mice. These results suggest that juvenile SDS exposure results in later onset of IBS-like symptoms.

## Introduction

Irritable bowel syndrome (IBS) is one of the most common functional gastrointestinal disorders. IBS is characterized by abdominal pain and altered bowel habits, including constipation, diarrhea or both, and is categorized according to the Rome IV criteria^1^. IBS patients often report a fear of incontinence in public spaces due to loose stools and fecal urgency, therefore IBS can affect quality of life^2^. The pathogenesis of IBS is heterogenous and complex. Psychological, physiological, and social factors, the immune system, gut microbiota, epithelial permeability, and altered brain–gut axis interactions have all been linked to IBS^3^. There has been a growing interest in the influence of psychological factors because external events have been shown to affect digestive function and common psychiatric comorbidities, such as anxiety disorders, in IBS^4^. Stressful life events, especially adverse events early in life, such as general trauma and abuse, are strongly associated with the development of IBS^5^. Psychiatric comorbidities and stressful conditions under the coronavirus disease 2019 pandemic also deteriorate IBS symptoms^6^.

Several IBS animal models have been developed, including models induced by the maternal separation, wrap restraint stress, heterotypic chronic stress, water avoidance stress, chemical irritants, infection, toxin, and repetitive colorectal distention^7–9^. Among them, neonatal maternal separation is a well-established model of IBS, mimicking early deprivation of maternal care in humans^10^. Neonatal maternal separation, in which pups are separated for 3 h daily during early postnatal days, is a form of trauma due to maternal neglect. This stress induces long-lasting IBS-like alterations in rats, such as visceral hypersensitivity, enhanced intestinal motility in response to acute stress, changes of the hypothalamic–pituitary–adrenal axis, and an increase in mast cells and enterochromaffin cells in gastrointestinal tracts^10,11^. However, some reports have demonstrated that maternal separation paradigms in mice are often inconsistent because of the difficulty to induce consistent and robust behavioral alterations in adults^12,13^. This suggests that the degree of physical stresses in maternal separation might be insufficient to sustain the effects of psychological stress in adult mice. Therefore, there is a need for more reliable mouse models to investigate the pathogenesis of IBS.

A social defeat stress (SDS) model, which subjects a mouse to repeated social subordination by an aggressor male mouse, is used as a chronic stress model^14^. SDS induces profound and stable behavioral changes such as anxiety- and depression-like behavior, including social avoidance^14,15^. There is accumulating evidence that certain types of stress in childhood can have long-lasting consequences on adult behavior^16,17^. Social behavior impairment induced by SDS exposure in juvenile mice persisted for 5 weeks^18^. Therefore, exposing juvenile mice to SDS may be useful for studying the pathogenesis of chronic stress-related psychiatric impairments in adolescents with early adverse experiences.

We speculate that juvenile SDS mimicking early life adverse events may be useful for studying the underlying mechanisms of IBS. The aims of the current study were to investigate the effects of juvenile SDS on adult mice. We conducted social avoidance, tail suspension, forced swim, and elevated plus maze tests, and immunohistochemical and fecal bacteria analyses in the colon 5 weeks after SDS exposure. To gain further insight into the relationship between juvenile SDS and secondary stressors in adults, we investigated the effect of restraint stress on peripheral and central factors in adults. Our results suggest that juvenile SDS can be used as an experimental model for studying early in life adverse events associated IBS.

## Results

### Social avoidance behavior was identified at 5 weeks after exposure to juvenile SDS

**Figure 1:**
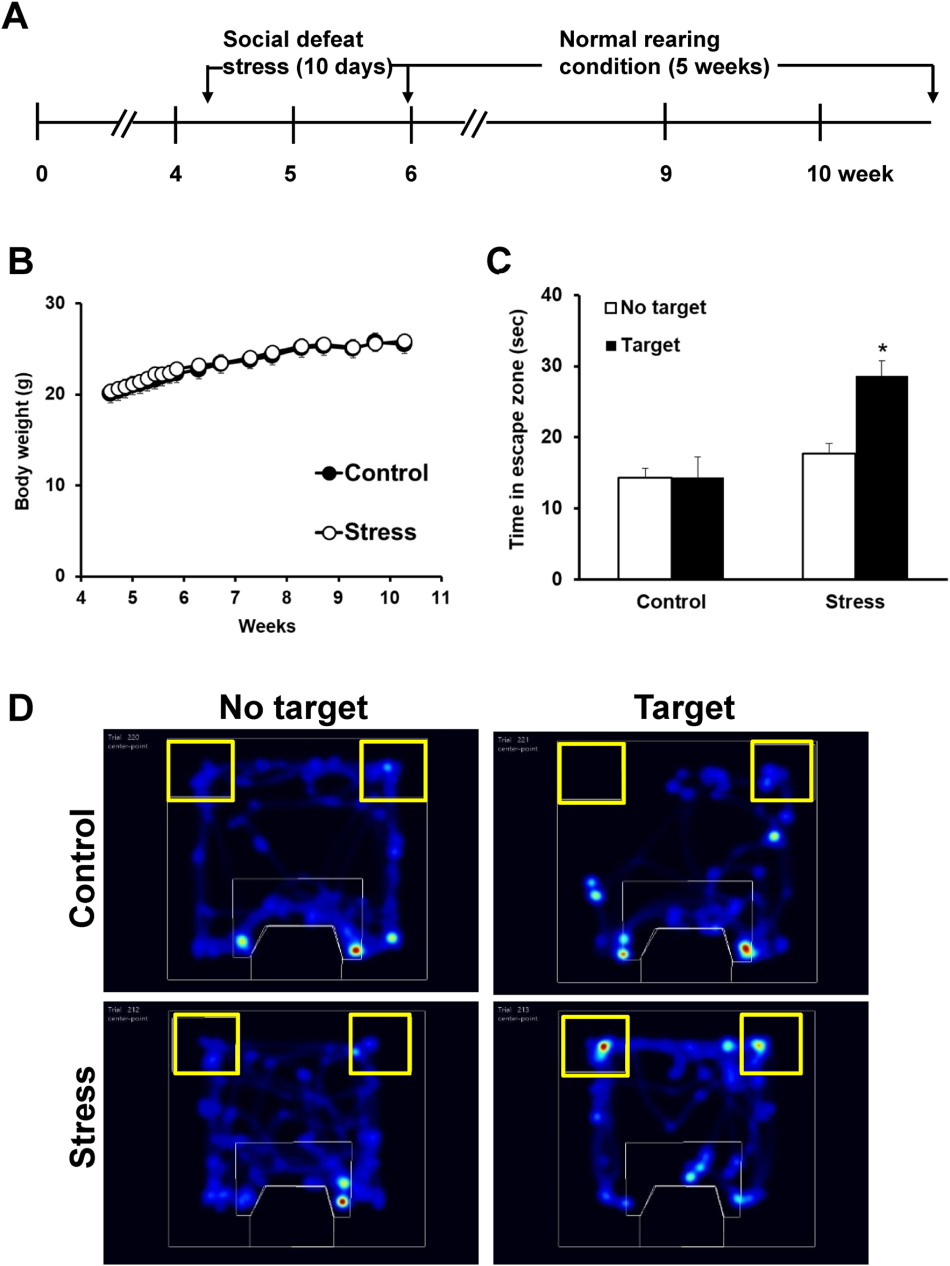
Social avoidance behavior was identified at 5 weeks after the exposure of juvenile social defeat stress (stress). (**A**) Protocol of juvenile SDS. (**B**) Body weight change during the juvenile stress and post-stress periods. (**C**) Duration of time spent in the escape zone (yellow square) with (target) or without (no target) aggressor mouse. (**D**) Representative results of heat maps in social interaction test with or without aggressor mouse. Data are presented as the mean ± SEM (n = 8 mice per group). *p < 0.05 for comparison with the corresponding no target.

We first investigated whether the effects of juvenile SDS were sustained for 5 weeks after the exposure to stress. Juvenile SDS did not affect body weight either during the SDS period or during the control rearing period (Fig. 1B). During the first session of the social interaction test (no target), juvenile SDS (stress) mice were similar to control (control) mice in the general pattern of arena occupancy (Fig. 1C,D). However, in the presence of an aggressor mouse (target), the stress group spent significantly more time in the escape zone (28.6 ± 2.2, *p* = 0.0123) compared with the control group (17.7 ± 1.5).

### Juvenile SDS induces anxiety- and depressive-like behaviors

**Figure 2:**
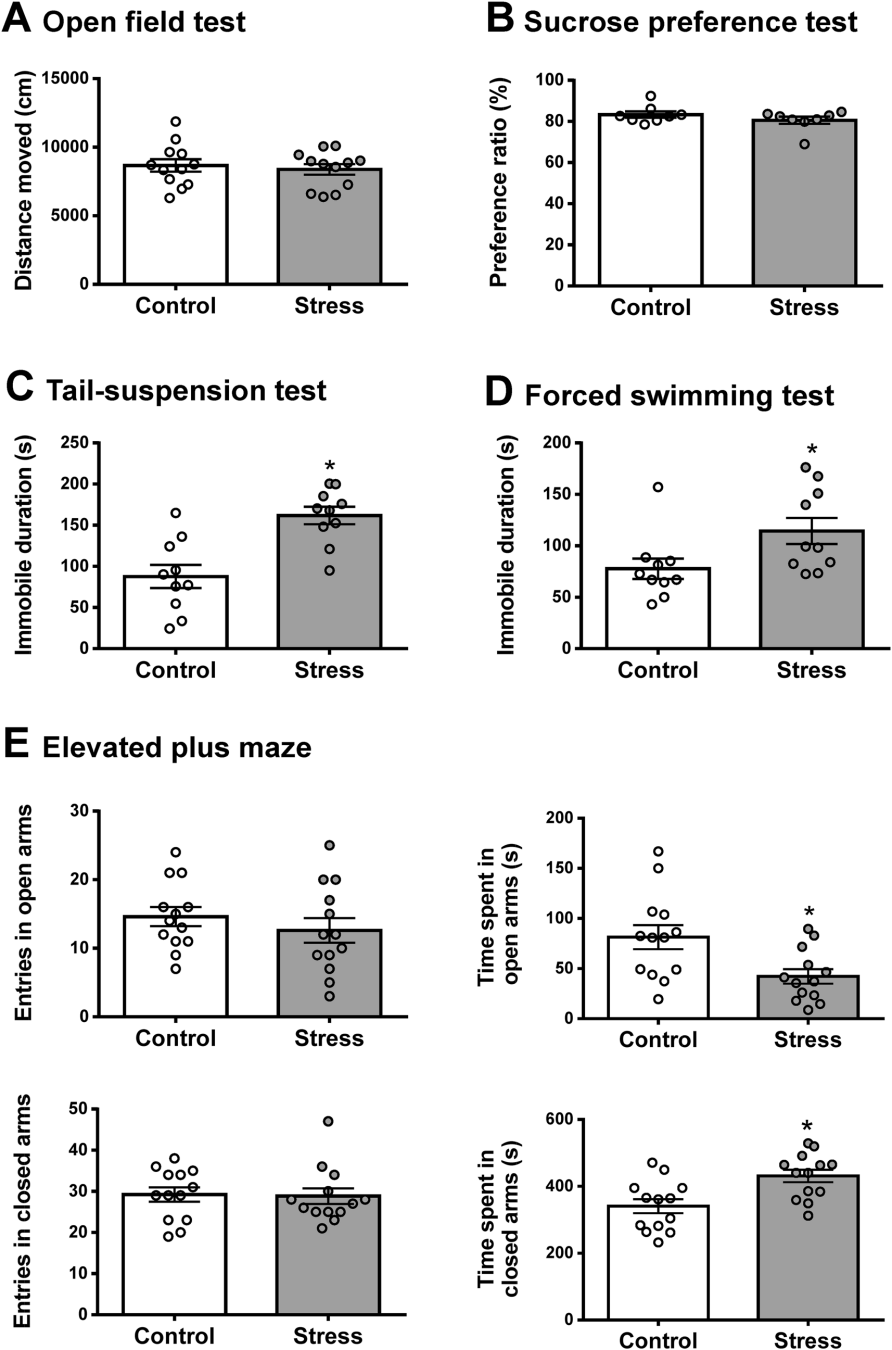
Juvenile SDS induced depression- and anxiety-like behaviors. The behaviors of control and juvenile SDS mice (stress) in open field (**A**), sucrose preference (**B**), tail suspension (**C**), forced swimming (**D**) and elevated plus maze (**E**) tests. Data are presented as the mean ± SEM (n = 8–13 mice per group). *p < 0.05 compared with the control group.

In the open field test, there was no significant difference in the traveled distances between control and juvenile SDS (stress) mice (Fig. 2A). There was no difference in the sucrose preference ratio between control and stress mice (Fig. 2B). To assess depression-like behaviors in juvenile SDS-treated mice, we performed the tail-suspension and forced swimming tests (Fig. 2C,D). Mice exposed to juvenile SDS exhibited a significant increase in immobility time (161 ± 11, *p* = 0.0006 and 114 ± 12, *p* = 0.0349) compared with control mice (87.7 ± 14.1 and 77.7 ± 4.8) in tail-suspension and forced swimming tests, respectively. Additionally, mice exposed to juvenile SDS exhibited a significant decrease in the time spent in the open arms (42.3 ± 7.1, *p* = 0.0099) compared with control mice (91.3 ± 17.1) (Fig. 2E). Mice exposed to juvenile SDS exhibited a significant increase in the time spent in the closed arms (431 ± 20, *p* = 0.0037) compared with control mice (340 ± 21) (Fig. 2E). There were no significant differences in the number of entries into the open arms and closed arms between the stress and control groups (Fig. 2E).

### Juvenile SDS increased visceral sensitivity to colorectal distension

**Figure 3:**
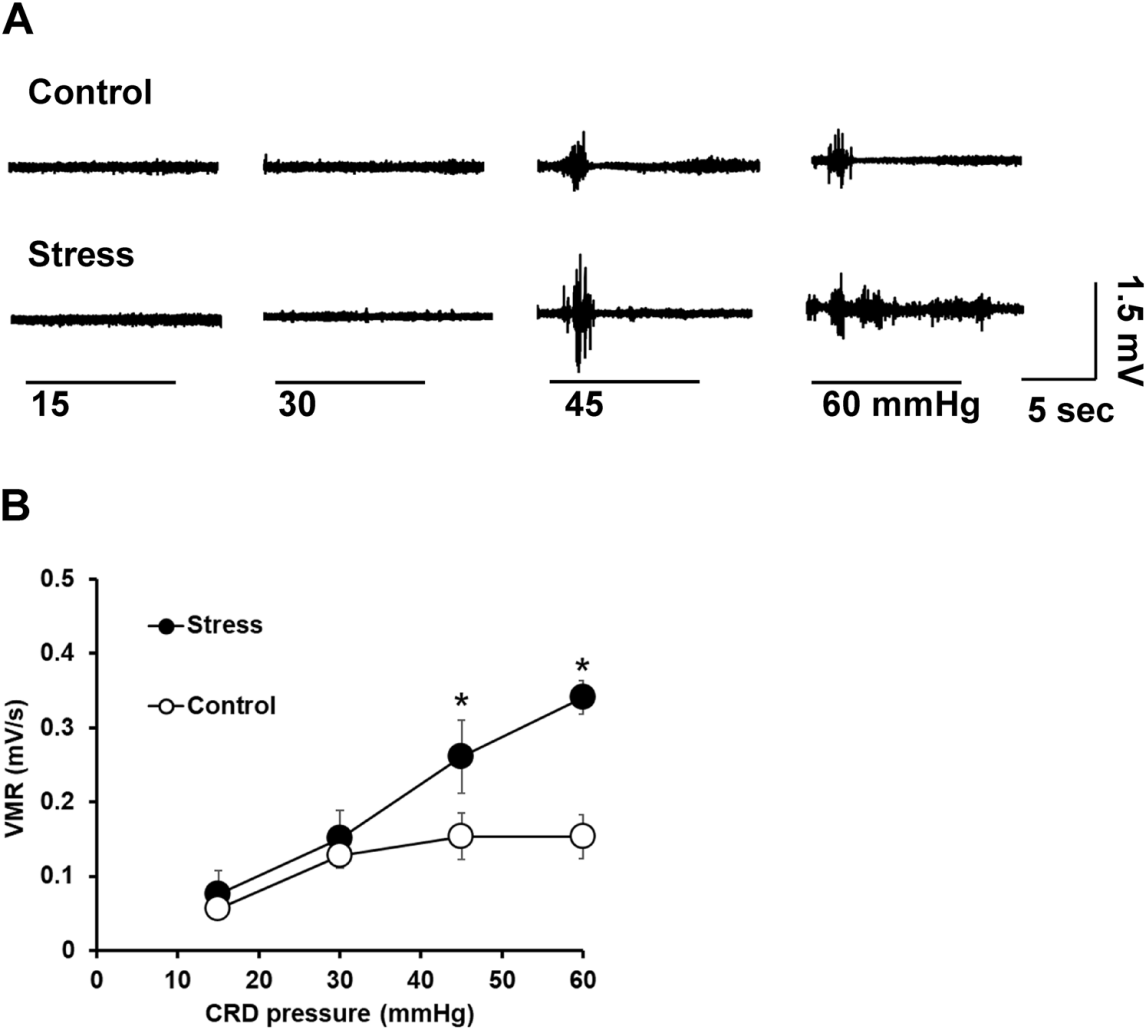
Juvenile SDS induced colonic visceral hyperalgesia. (**A**) Representative electromyographic recordings of control and SDS model (stress) mice. (**B**) Visceromotor response (VMR) to colorectal distension (CRD) in control and SDS model mice. Data are presented as the mean ± SEM (n = 6–8 mice per group). *p < 0.05 compared with the control group.

We next investigated the effect of juvenile stress on visceral hyperalgesia (Fig. 3). The amplitude of the electromyographic recordings indicated a significant stimulus effect (F = 7.1, *p* = 0.0007). There was no significant group effect (F = 2.9) or group × stimulus interaction effect (F = 2.3). The visceromotor response to colorectal distension was significantly increased in mice exposed to juvenile SDS compared with that in control mice at 45 (*p* = 0.0454) and 60 mmHg (*p* = 0.0293), which is indicative of the development of visceral hyperalgesia.

### Juvenile SDS increased the number of 5-HT-positive cells and CGRP-positive neurons in colon

**Figure 4:**
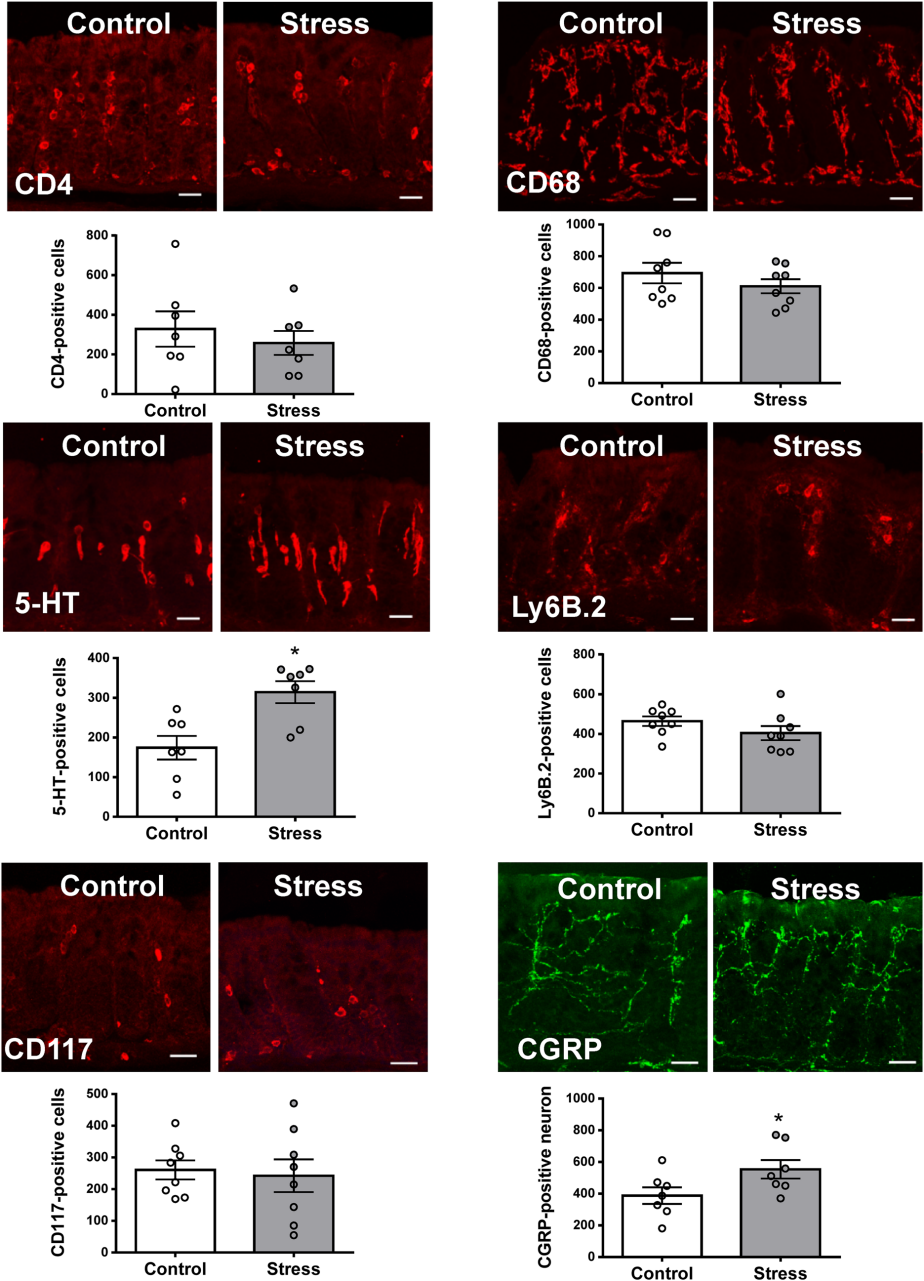
Juvenile SDS increased the number of 5-HT-positive cells and CGRP-positive neurons. Representative images of immunohistochemistry for CD4-, CD68-, 5-HT-, Ly6B.2-, and CD117-positive cells and CGRP-positive neurons in the colonic mucosa of control and stress mice. The number of CD4-, CD68-, 5-HT-, Ly6B.2-, and CD117-positive cells, and CGRP-positive neurons per 10^6^ μm^2^ were counted. Data are presented as the mean ± SEM (n = 7, 8 mice per group). *p < 0.05 compared with the control group. Scale bars, 50 μm.

We next investigated the number of cells that expressed of CD4, CD68, 5-HT, Ly6B.2, and CD117, and CGRP-positive neurons in the colonic mucosa of control and stress mice (Fig. 4). Juvenile SDS exposure significantly increased the number of 5-HT-positive cells (*p* = 0.0049) and CGRP-positive neurons (*p* = 0.0466) in the colon, but not the number of CD4-, CD68-, Ly6B.2-, or CD117-positive cells in comparison with the control group. There was no significant difference in the histological score between the control and stress groups (Supplementary Fig. 1A). Alcian blue and periodic acid schiff staining were used to highlight the goblet cell population (Supplementary Fig. 1B). The number of goblet cells per trench was similar between the groups.

### Gut microbiota was largely similar between the control and juvenile SDS groups

**Figure 5:**
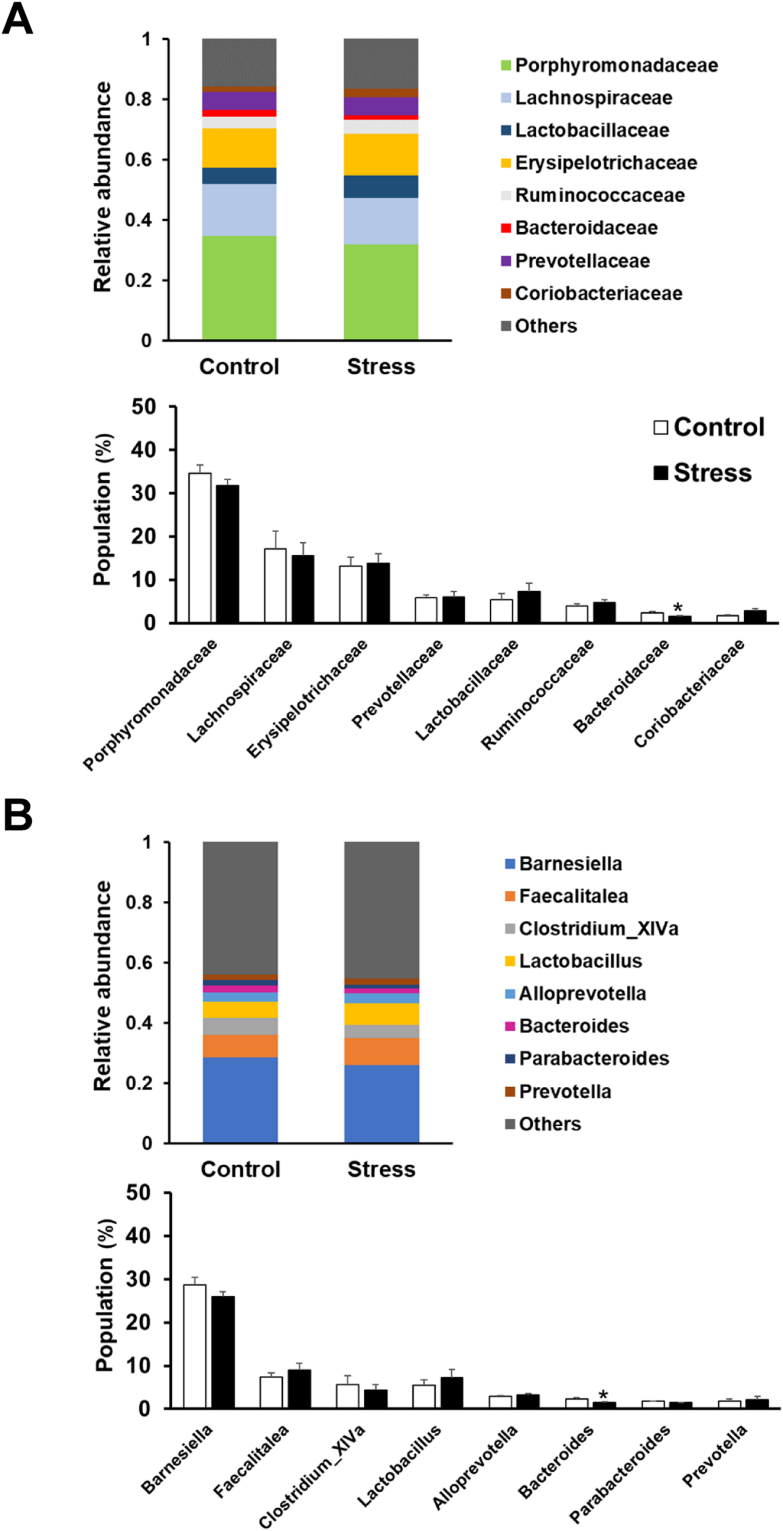
Composition of gut bacterium at the levels of family (**A**) and genus (**B**) in the control and juvenile SDS (stress) mice. Microbiome analysis of fecal samples for each bacterial genus was performed using next generation sequencing of bacterial 16S rDNA. The relative abundance (upper panel) and population (lower panel) of the top eight gut bacterium in control and juvenile SDS mice stool samples. Data are presented as the mean ± SEM (n = 8–10 mice per group). *p < 0.05 compared with the control group.

Next, we investigated the influence of juvenile SDS on gut microbiota for the eight most abundant (having a relative abundance > 1%) bacterial family (Fig. 5A) and genus (Fig. 5B) levels, in control and juvenile SDS (stress) mice. There were no considerable differences between the control and stress groups. *Bacteroidaceae* (*p* = 0.0102) at the family level and *Bacteroides* (*p* = 0.0159) at the genus level were significantly less abundant in the juvenile SDS group than in the control group (Fig. 5B). The other major genera showed no differences in abundance between the groups.

### Restraint stress increased fecal pellet output, plasma corticosterone concentration, and serotonin content in juvenile SDS mice

**Figure 6:**
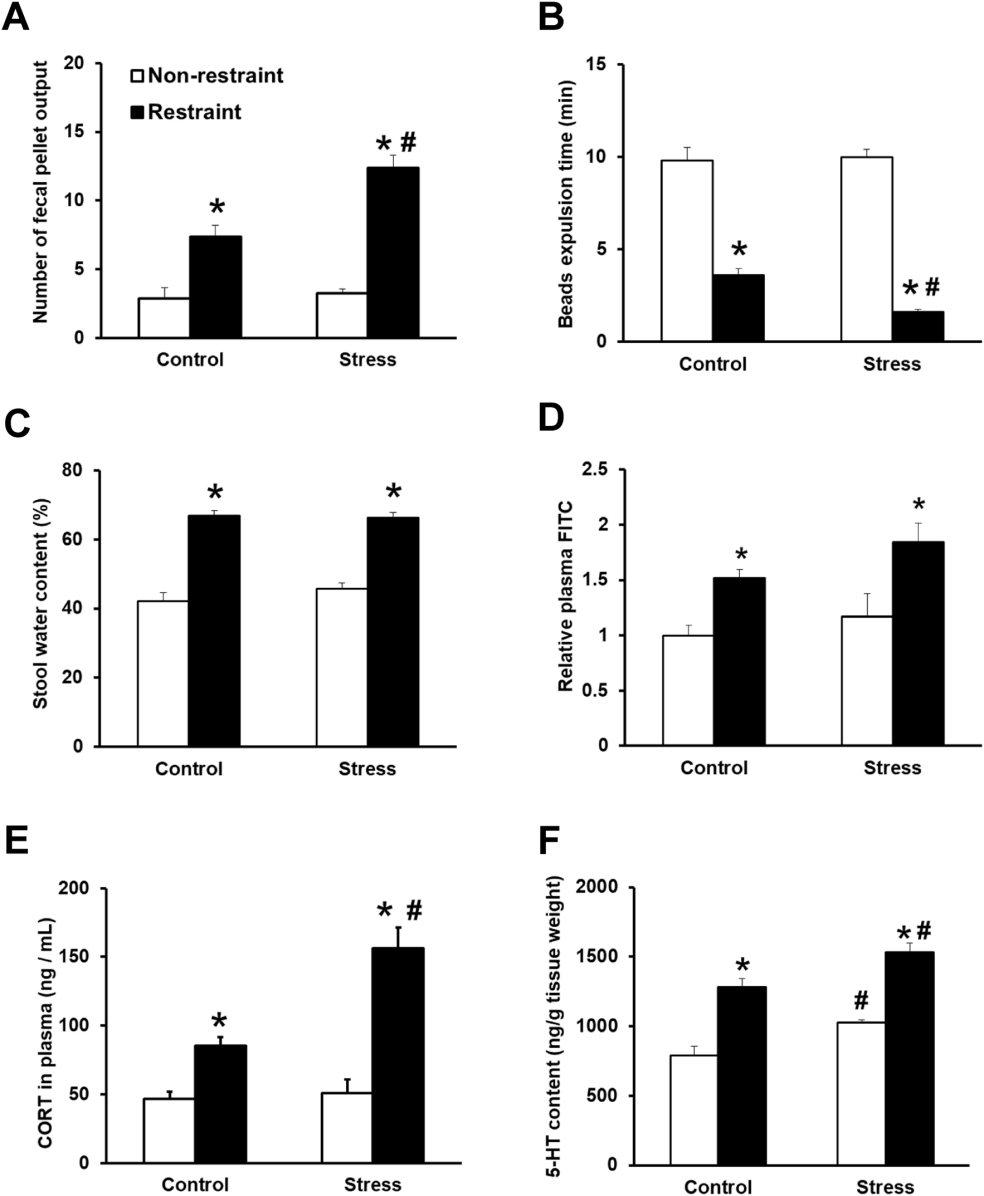
Juvenile SDS exacerbated stress responses in the restraint condition. Fecal pellet output number (**A**), bead expulsion time (**B**), stool water content (**C**), relative plasma FITC content (**D**), plasma corticosterone (CORT) levels (**E**), and 5-HT content (**F**)of control and juvenile SDS (stress) mice in the non-restraint and restraint stress conditions. Data are presented as the mean ± SEM (n = 8 mice per group). *p < 0.05 for comparison with the non-restraint group. ^#^*p* < 0.05 for comparison with the control group in the restraint condition.

To investigate the vulnerability to acute severe stress in adults, we evaluated the number and characteristics of fecal pellets, stress susceptibility, and intestinal permeability in juvenile SDS (stress) and control mice under restraint stress conditions. The number of fecal pellets excreted during restraint stress was significantly increased in stress group (12.4 ± 0.9, *p* = 0.0211) compared with the control group (7.38 ± 0.81, Fig. 6A). The bead expulsion time during restraint stress was significantly decreased in stress group (1.60 ± 0.17, *p* = 0.0329) compared with the control group (3.61 ± 0.72, Fig. 6B). There was not significant difference between the groups on stool water content after the restraint stress (Fig. 6C). To assess stress susceptibility, we examined the plasma corticosterone level (Fig. 6E). There were no significant differences in the basal corticosterone level between the stress and control groups. In animals that received the restraint stress treatment, the corticosterone concentration in plasma was significantly increased in the stress group (156 ± 15, *p* = 0.0105) compared with the control group (85.6 ± 5.2). The plasma levels of FITC dextran in the control (*p* = 0.0311) and stress groups (*p* = 0.0465) were significantly increased by restraint stress exposure compared with that in the corresponding non-restraint groups (Fig. 6D). However, there was no significant difference between the stress and control groups in the restraint condition (Fig. 6D). The colonic content of 5-HT in the control (*p* = 0.0003) and stress groups (*p* < 0.0001) was significantly increased by restraint stress exposure compared with that in the corresponding non-restraint groups (Fig. 6F). The 5-HT content in stress group (1529 ± 69, *p* = 0.0313) was significantly higher than that in the control group (1280 ± 61) in the restraint condition (Fig. 6F).

### Juvenile SDS affected noradrenergic pathway in amygdala during acute severe stress in adults

**Figure 7:**
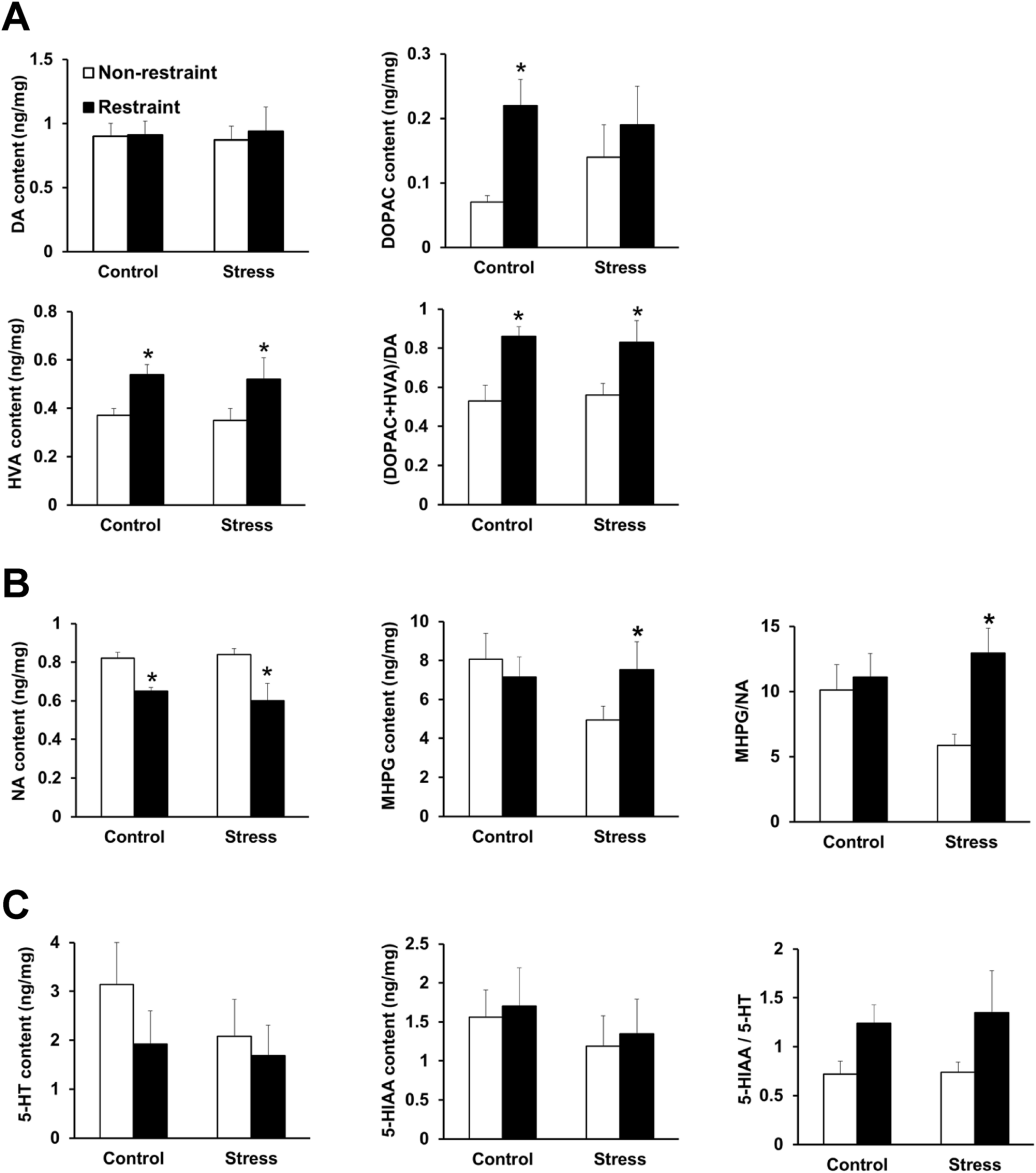
Monoamine and metabolite levels in the amygdala of control and juvenile SDS (stress) mice in the non-restraint and restraint stress conditions. (**A**) Dopamine (DA), homovanillic acid (HVA), and 3,4-dihydroxyphenylacetic acid (DOPAC) content, and (DOPAC + HVA)/DA ratio in control and juvenile SDS mice in the non-restraint and restraint stress conditions. (**B**) Noradrenaline (NA) and 3-methoxy-4-hydroxyphenylglycol (MHPG) content and MHPG/NA ratio in control and juvenile SDS mice in the non-restraint and restraint stress conditions. (**C**) Serotonin (5-HT) and 5-hydroxyindole acetic acid (5-HIAA) content and 5-HIAA/5-HT ratio in control and juvenile SDS mice in the non-restraint and restraint stress conditions. Data are presented as the mean ± SEM (n = 8 mice per group). *p < 0.05 for comparison with the non-restraint group.

Finally, we investigated the levels of monoamines and their metabolites in six brain regions (amygdala, frontal cortex, hippocampus, hypothalamus, limbic, and striatum) in the control and juvenile SDS (stress) groups with or without restraint stress (Supplementary Tables 1–3). Of the six regions, the effect of juvenile SDS and restraint stress on the levels of monoamines and their metabolites was especially pronounced in the amygdala (Fig. 7). Juvenile SDS alone did not affect basal levels of dopamine, noradrenaline, 5-HT, 3,4-dihydroxyphenylacetic acid (DOPAC), homovanillic acid (HVA), 3-methoxy-4-hydroxyphenylglycol (MHPG), or 5-hydroxyindole acetic acid (5-HIAA) in the amygdala. Restraint stress had no effect on dopamine in the control and stress groups. Restraint stress significantly increased HVA (*p* = 0.0082 and *p* = 0.0009) and the DOPAC + HVA/dopamine ratio (*p* = 0.0103 and *p* = 0.0263) compared with the non-restraint stress groups in both the control and stress groups, respectively (Fig. 7A). Restraint stress significantly decreased noradrenaline compared with the non-restraint group in both the control (*p* = 0.0002) and stress groups (*p* = 0.0005) (Fig. 7B). There was an interaction between juvenile SDS and acute restraint stress on noradrenaline metabolism in the amygdala. Restraint stress significantly increased noradrenaline metabolite MHPG (*p* = 0.0494) and the MHPG/noradrenaline ratio (*p* = 0.0142) compared with non-restraint in the stress group (Fig. 7B). These alterations were not observed in the control group. Restraint stress had no effect on 5-HT and its metabolite in the control and stress groups in the amygdala (Fig. 7C).

## Discussion

Early life stress is a significant risk factor for development and maintenance of IBS. There are multiple ways to measure the outcome in an experimental animal model of IBS, including physiologic and reflex responses, spontaneous behaviors, visceral pain-directed complex behaviors, or brain responses^19^. The major findings of this study are as follows. Firstly, we confirmed that juvenile SDS caused anxiety- and depression-like behaviors and visceral hypersensitivity in adult mice. Secondly, juvenile SDS mice showed vulnerability to the effects of acute restraint stress in adulthood. Thirdly, the interaction between juvenile SDS and acute restraint stress affected noradrenaline metabolism in the amygdala.

Multiple factors such as duration of physical contacts, aggressive behavior, types of food, body water content, affect body weight characteristics during the SDS period^20^. A previous study reported that defeated mice showed reduced body weight during a 10-day SDS paradigm^21^. However, other studies reported that defeated mice showed increased body weight compared with a control group during the stress period^22^. In the present study, juvenile SDS did not affect body weight either during the SDS period or during the control rearing period. Therefore, body weight may not be a reliable indicator of SDS. The SDS model is well established in adult mice to induce anxiety- and depression-like behaviors^23^. Mouri et al. reported that social avoidance behavior in mice exposed to SDS as juveniles is more persistent than these behaviors in mice exposed to SDS as adults^18^. In the present study, juvenile SDS induced social avoidance behavior in a social interaction test, decreased the duration of time spent in open arms in the elevated plus maze test, and increased immobility in the tail-suspension and forced swimming tests at 5 weeks after the exposure to juvenile SDS. There were no significant differences in locomotor activity in the open field test between control and juvenile SDS mice. These results suggest that juvenile SDS provoked social aversion and anxiety- and depression-like behaviors, but did not affect spontaneous motor activity in adults.

The pathogenesis of visceral hypersensitivity is a complex process and not well understood^24^. Chronic visceral pain, which can be mediated by peripheral and central pathways, is a typical symptom of IBS^24^. SDS in adulthood induces visceral hypersensitivity in C57BL/6 mice 24 h after the last SDS session^25^. Our data indicate that the lack of significant statistical interaction of group effect or group × stimulus interaction effect. Post hoc tests revealed that the visceromotor responses of juvenile SDS mice was significantly higher than control mice only at 45 and 60 mmHg. These results suggest that SDS during childhood affected visceral sensitivity 5 weeks after the last SDS session. It has been previously shown that neonatal maternal separation induces an increase of CGRP expression and 5-HT content, and contributes to visceral hypersensitivity in adults^26^. Increased CGRP release in the colon of mice exposed to water avoidance stress induced visceral hyperalgesia in the mice^27^. Furthermore, social stress induces TRPV1, which colocalizes with CGRP-dependent afferent nerve activity in mouse urinary bladder^28^. In the present study, juvenile SDS mice showed vulnerability to acute restraint stress in adulthood and had an increased number of sensory neurons and 5-HT content in the colon. These results suggest that the visceral hypersensitivity in adult mice induced by juvenile SDS is possibly related to complex central and peripheral factors.

Accumulating evidence suggests that maternal separation and SDS affect gut microbiota associated with psychological and gastrointestinal functional disorders^29,30^. We investigated gut microbiota profiles 5 weeks after juvenile SDS exposure. Although the profiles were largely similar between the control and juvenile SDS groups, we found significant reductions of *Bacteroidaceae* and *Bacteroides* in the juvenile SDS mice compared with control mice. Qu et al. has also reported marked reductions of *Bacteroides* in SDS mice^29^. Low levels of *Bacteroides* are associated with the pathogenesis of depression^30^. Our results indicate that juvenile SDS did not strongly affect gut microbiota. Previous studies reported that chronic SDS in adulthood showed the largest changes in gut microbiota^31,32^. In the present study, we were unable to clearly identify the effect of juvenile SDS on gut microbiota profiles in adulthood. However, the alterations of *Bacteroidaceae* and *Bacteroides* might play a role in psychological and gastrointestinal dysfunction induced by juvenile SDS.

Many IBS patients have difficulty coping with stressful conditions and suffer from anxiety, depression or panic disorder^4^. To analyze coping ability during severe stress in adults, we investigated the effect of restraint stress on intestinal motility, permeability, corticosterone and 5-HT levels in colon, and the levels of monoamines and metabolites in six brain regions. Interestingly, introduction of restraint stress in the juvenile SDS group provoked significant gastrointestinal hypermotility, increased corticosterone and 5-HT in the colon and increased noradrenaline turnover in the amygdala compared with control mice, suggesting a vulnerability to acute stress in adulthood.

Characterization of fecal pellet output is a relevant readout to assess gastrointestinal function primarily related to motility, secretion, and permeability^33^. It has previously been shown that early life stress in rats increases fecal pellet output following water avoidance stress or exposure to a novel environment^34^. Several studies have demonstrated that the exposure of animals to various acute stressors, such as water avoidance or maternal separation, can affect intestinal barrier function^35^. We found that juvenile SDS alone did not affect intestinal integrity, secretion, or intestinal motility and permeability in adults in the non-restraint condition. However, combined with juvenile SDS, restraint stress increased the fecal pellet output number and colonic motility, but did not affect water content or intestinal permeability when compared with restraint stress in the control group. These results suggest that juvenile SDS induced intestinal motility dysfunction under an acute stress condition in adulthood.

In a previous study, postprandial 5-HT was significantly higher in patients with diarrhea-dominant IBS than in healthy volunteers^36^. In experimental animals, altered 5-HT signaling contributes to visceral hypersensitivity and bowel habits including motility and secretion^37^. Results from experimental studies have shown that early life stress induces enterochromaffin cell hyperplasia in the gut of adult animals^38^. A previous study has reported that 5-HT levels in the colon are increased in neonatal maternal separation rats^26^. Additionally, it has been shown that combined neonatal maternal separation and acute water avoidance stress induces visceral hypersensitivity and motility dysfunction, accompanied by an increase in 5-HT content and enterochromaffin cell number in rat colon^39^. In a report by De Palma et al., there was no statistically significant effect of neonatal maternal separation on 5-HT levels in the hippocampus and amygdala^40^. In this study, juvenile SDS significantly increased 5-HT levels in the colon compared with 5-HT in control mice, in both the non-restraint and restraint stress groups. However, juvenile SDS did not affect 5-HT and its metabolism in the examined brain regions. These results are consistent with previous studies in neonatal maternal separation^39,40^ and suggest that juvenile SDS induces long term 5-HT alterations in the periphery.

The hypothalamic–pituitary–adrenal axis is the physiological system involved in coping with stressors, and matures during adolescence^41^. It is strongly linked to both anxiety and depression disorders and regulates circulating levels of corticosterone. In the present study, there was no significant difference in basal corticosterone levels between the juvenile SDS and control mice. Restraint stress exposure for 1 h increased corticosterone levels in both juvenile SDS and control mice. Interestingly, the acute stress during adulthood significantly increased the corticosterone level in juvenile SDS mice compared with that in control mice. These results suggest that mice exposed to SDS as juveniles could not cope well with acute stress in adulthood and displayed higher hypothalamic–pituitary–adrenal axis activation.

Release of monoamines in the frontal cortex, hippocampus, hypothalamus, amygdala, striatum, and limbic regions has been associated with anxiety and depression^42^. Among these regions, our results suggest that the amygdala is involved in the interaction between juvenile SDS and acute restraint stress. The amygdala is a key component of the neural network that determines the emotional significance of external events and organizes the behavioral response to emotionally significant events^43^. In the present study, we found that restraint stress significantly decreased noradrenaline and increased the MHPG/noradrenaline ratio in juvenile SDS mice compared with control mice. It has been shown that noradrenaline in the bed nucleus of the stria terminalis (also called extended amygdala), which plays important role in the emotional processing in combination with amygdala, affect gastric emptying and small intestine transit through β-adrenergic receptors^44^. The interaction between juvenile SDS and acute restraint stress was observed especially in the noradrenaline metabolite MHPG and the MHPG/noradrenaline ratio in the amygdala. It has been previously reported that both restraint stress and SDS in adults increases MHPG accumulation in the amygdala^45^. These results suggest that the extent of noradrenaline utilization, reflected by elevated MHPG, was increased by the acute restraint stress particularly in juvenile SDS mice. Noradrenaline is usually released in the amygdala in response to the various stimuli^46^. Therefore, the increased noradrenaline turnover ratio suggested that noradrenaline released from the presynaptic neurons were rapidly metabolized into MHPG. It has been previously reported that noradrenaline transporter-deficient mice show social interaction impairments and depression-like behaviors in SDS and restraint stress models^47^. Therefore, it is possible that the increase of noradrenaline turnover is linked to the behavioral alterations observed during adulthood in juvenile SDS mice. Dopamine neurons in the mesocorticolimbic area are activated by stressful stimuli, similar to noradrenaline neurons^48,49^. Therefore, we calculated the ratio between DOPAC + HVA/dopamine as an index of acute restraint stress. Our results indicated that there was no difference in the ratio between the juvenile SDS group and normal group, which suggests that juvenile SDS had no effect on mesocorticolimbic dopamine neurons.

There are multiple limitations that must be considered when interpreting the results of this study using juvenile SDS as a model of early life stress associated with IBS. Firstly, further characterization of the effects of juvenile SDS during adolescence, such as on peripheral and central sensitization and immune responses, will be required to validate this paradigm as an early life stress-induced IBS model. Secondly, we did not investigate the relationship between juvenile SDS and IBS-like symptoms using drugs for IBS treatment. In this regard, future studies should consider using current IBS drugs, such as anti-diarrheal agents and anti-depressants, on the juvenile SDS model. Thirdly, further characterization of SDS’s effects on gut microbiota (for example, by performing a fecal bacteria analysis in the colon immediately after juvenile SDS or restraint stress exposure) would provide additional insight. Finally, further studies investigating the differences of sex and developmental stages in mice exposed to SDS are needed to better understand IBS-like alterations on the gastrointestinal system and brain.

**Figure 8:**
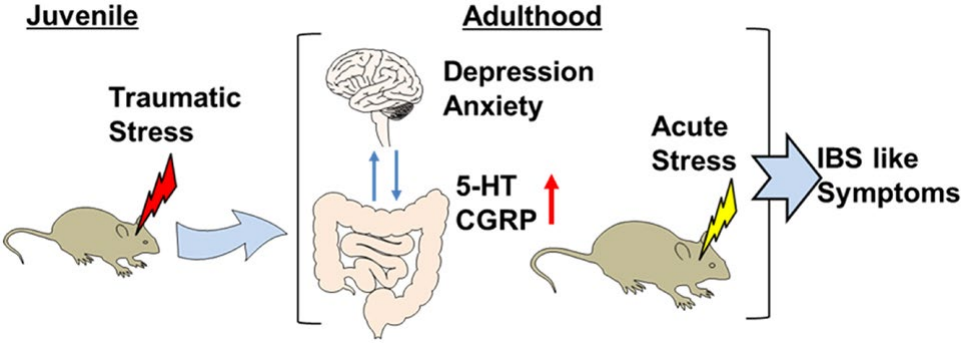
The schematic diagram of the juvenile SDS induced IBS-like symptoms in adulthood. Juvenile traumatic SDS caused anxiety-/depression-like behaviors, and an increase of CGRP/5-HT expression in large intestine on adulthood. Juvenile SDS mice showed vulnerability against acute restraint stress and IBS-like symptoms such as visceral hypersensitivity, psychiatric symptoms, and motility disorder on adulthood. Image was generated using Motifolio illustration tool kit (Motifolio Inc., Ellicott City, MD, USA).

The present findings show that the juvenile SDS model demonstrates the main features of early life stress-associated IBS, such as visceral hypersensitivity, psychiatric symptoms, motility disorder and vulnerability to acute severe stress in adulthood (Fig. 8). These results suggest that juvenile SDS exposure results in later onset of IBS-like symptoms. Therefore, this model may be useful for studies on early life stress-associated IBS.

## Materials and methods

All methods were carried out in accordance with relevant guidelines and regulations.

### Animals

Male C57BL/6 mice (4 weeks old) and male adult CD1 (ICR) mice (13–15 weeks old) were purchased from Japan SLC Inc. (Shizuoka, Japan). All mice were maintained in plastic cages with free access to food and water, and housed at 22 ± 1 °C with a 12 h light/dark cycle. This study was carried out in strict accordance with ARRIVE for reporting experiments involving animals^50^. The protocols were approved by the committee on the Ethics of Animal Research of Kyoto Pharmaceutical University (Permit numbers: 19–011). The number of animals was kept to the minimum necessary for meaningful interpretation of the data, and all efforts were made to minimize animal suffering.

### Juvenile SDS model

The SDS model has been employed relatively in male rodents because of the difficulty of initiating attack behavior directed toward female mice^51^. We therefore conducted the SDS paradigm in juvenile male mice. C57BL/6 male mice weaned after 4 weeks of age show increased behavioral stability compared with mice weaned at 3 weeks of age^52^. Therefore, we purchased 4-week-old mice and began experiments after a 3-day habituation period. This condition did not strongly affect the growth of the mice (Fig. 1B). The procedure of juvenile SDS was performed as reported previously with modifications (Fig. 1A)^53^. Briefly, each 4-week-old C57BL/6 mouse was exposed to a different CD1 aggressor mouse once a day for 10 min. After the interaction, the mice were returned to their home cages and kept isolated until SDS on the next day. Mice were subjected to this procedure for 10 consecutive days. The pairs of defeated and aggressor mice were randomized daily to minimize the variability in aggressiveness between the aggressor mice. To avoid habituation to the presence of C57BL/6 mice, all aggressor mice were screened for aggressive behavior before their use in the consecutive social defeat experiments. The control mice were housed in a similar cage without interaction with CD1 mice. Defeated and control mice were housed 4–5 mice per cage for 5 weeks after the last exposure to defeated stress and then investigated each experiment. Social interaction was evaluated as previously reported^53^. The time spent in the escape zone (corner zone, Fig. 1D, yellow line) was measured and social avoidance behavior was recorded using EthoVision XT Software (Noldus, Wageningen, Netherlands).

### Assessment of visceromotor responses to colorectal distension

Assessment of visceromotor responses to colorectal distension was performed at 5 weeks after the exposure to juvenile SDS. As a visceral stimulus, mechanical distensions of the rectum were performed by pressure-controlled air inflation of a flexible polyethylene balloon connected to an electronic distension device (Distender Series II barostat, G&J Electronics, Willowdale, ON, Canada). The balloon was lubricated, inserted intra-anally and positioned 5 mm proximal to the anus. The visceromotor responses to colorectal distension were quantified by electromyographic recordings of abdominal wall muscle activity. Mice were challenged with distending pressures of 15, 30, 45, and 60 mmHg, with two 10-s trials at each pressure and a 2-min recovery period between distensions. Data were imported into 8-channel analyzer software (Starmedical, Tokyo, Japan) for analysis. Representative raw electromyographic recordings are depicted in mV. The electromyographic baseline activity during the 10-s before stimulation was subtracted from the 10-s of each reflex response.

### Immunohistochemistry

Immunohistochemical procedures were performed as previously described^54^. Sections were probed for 40 h at room temperature with rat anti-CD4 (1:500, BD Bioscience, San Jose, CA, USA), rat anti-CD68 or rat anti-Ly6B.2 (1:1,000, AbD Serotec, Bio-Rad, Raleigh, NC, USA), goat anti-5-HT (1:5000, Immunostar, Hudson, WI, USA), rat anti-CD117 (1:500, R&D Systems, Minneapolis, MN, USA), or sheep anti-calcitonin gene-related peptide (CGRP) antibody (1:4000, Enzo Life Sciences, Farmingdale, NY, USA). After washing in PBS, sections were incubated for 4 h at room temperature with corresponding secondary antibody. Quantitative determinations were made from three random locations for each mouse. For analysis, the number of CD4-, CD68-, Ly6B.2-, 5-HT-, and CD117-immunopositive cells, and CGRP-immunopositive neurons were counted per 10^6^ µm^2^ area of tissue.

### Behavioral test

For the open field test, each mouse was placed in the center of a circular open field chamber as reported previously^55^. Activity in the open field chamber was video recorded for 30 min and the distance of the movement was analyzed using EthoVision XT Software. The tail suspension test and forced swim test were performed in accordance with previously described methods^56^. Each mouse was suspended by its tail with tape, and the period of immobility was measured by a trained observer. During the 6-min test session, the last 4 min were recorded and analyzed by a video camera. Immobility was defined as the absence of any limb or body movements, except those caused by respiration. For the forced swimming test, each mouse was placed in a glass cylinder that contained water at 22 °C, and the period of immobility was recorded and analyzed using a video tracking system (EthoVision XT). The duration of the test was 6 min and immobility was measured during the last 4 min to facilitate comparisons with the tail suspension test. The elevated plus maze test was performed in accordance with previously described methods^57^. Briefly, each mouse was placed in the center of an elevated plus maze apparatus, facing an open arm, and allowed to freely explore it for 10 min. The number of entries and duration of time spent in the open and closed arms were measured using a video tracking system (EthoVision XT). For the sucrose preference test, each mouse was placed in a cage and given a choice between two bottles, one with 30 mM sucrose solution and the other with tap water. The position of the bottle was switched every 24 h. Intake from each tube was obtained by recording the weight of the fluid at the beginning and end of each 48-h test. Preference ratios were calculated as the intake of each solution divided by total intake and expressed as a percentage.

### Restraint stress

Experiments were performed at 5 weeks after the final day of SDS. Control and SDS mice were subjected to restraint stress by being placed individually into a restraint cage for 1 h. The number of fecal pellets excreted during the restraint stress was counted. The stool samples were dried for 24 h and water content (%) was calculated as follows: (stool wet weight − dry weight/stool wet weight) × 100.

### Bead expulsion test

Mice were fasted for 18 h before the experiments. A glass bead (approximately 3 mm in diameter) was inserted into the distal colon to a depth of 2 cm from the anus with a silicone tube. Bead expulsion time was measured in the non-restraint and restraint conditions.

### Measurement of corticosterone and 5-HT levels

Corticosterone in plasma and 5-HT content in the colon was analyzed by enzyme immunoassay using a enzyme immunoassay kit (Enzo Life Sciences) and (Beckman Coulter, Fullerton, CA, USA), respectively^58^.

### Intestinal permeability

FITC-dextran MW4000 (Sigma-Aldrich, St. Louis, MO) was administered to the mouse by gavage at a volume of 200 µL, using a stock solution at 50 mg/mL. After gavage, the mouse remained in the non-restraint or restraint condition. After 1 h, blood was obtained from the inferior vena cava. Samples were centrifuged (2000×*g* for 10 min), and the plasma concentration of FITC was measured the fluorescence intensity using a Varioskan Flash microplate reader (Thermo Fisher Scientific).

### Fecal bacteria analysis

Bacterial DNA was extracted from stool with NucleoSpin DNA Stool kit (MACHEREY–NAGEL GmbH & Co. KG, Dueren, Germany) according to the manufacturer's instructions. DNA was stored at − 80 °C until use. The V3–V4 region of bacterial 16S rRNA was amplified by PCR using specific primers. Each primer sequence was as follows. Forward primer: 5′-TCGTCGGCAGCGTCAGATGTGTATAAGAGACAGCCTACGGGNGGCWGCAG-3′, reverse primer: 5′-GTCTCGTGGGCTCGGAGATGTGTATAAGAGACAGGACTACHVGGGTATCTAATCC-3′. The amplicon was purified with AMPure XP beads. Then, a barcode sequence was added to each amplicon using Illumina Nextera XT Index kit v2 (Illumina, San Diego, CA, USA) for labeling and distinguishing the samples. The barcoded library was purified as described above, then diluted to 4 nmol/L in 10 mmol/L Tris–HCl (pH 8.0). Five microliters of each diluted sample were pooled and further diluted to 6 pmol/L using buffer from the respective sequencing kit. This sample DNA library was applied to a MiSeq Reagent Kit v3 (Illumina) and sequenced with 2 × 300-bp paired-end on a MiSeq, spiked with 5% PhiX control DNA (6 pmol/L). Annotation and calculation of obtained sequences were processed by 16S Metagenomics Database Creator v1.0.0.

### Quantification of monoamine contents in the brain

Mice were sacrificed immediately after the restrain stress, the brain was quickly removed and the frontal cortex, striatum, limbic region (brain region including nucleus accumbens, piriform cortex, nucleus of the limb of the diagonal band, and medial preoptic area), hypothalamus, hippocampus, and amygdala were dissected on an ice-cold glass plate. Hypothalamus, hippocampus, and amygdala were dissected on an ice-cold glass plate. The tissue samples were frozen at − 80 °C and stored until assayed. The concentrations of monoamines in the brain were determined by high-throughput liquid chromatography as reported previously^59^.

### Data and statistical analyses

Data are presented as the mean ± S.E.M. Statistical analyses were performed with GraphPad Prism 6.07 (GraphPad Software, La Jolla, CA, USA). Multiple groups were compared by two-way ANOVA. If the ANOVAs revealed a significant main effect or interaction between the factors, a post hoc Holm-Sidak test was performed. The Student’s t-test was used to compare two sets of data. *P*-values of < 0.05 were considered statistically significant.

## Supplementary Information

Supplementary Figure.

Supplementary Tables.
