# Supplementary material for: Juvenile social defeat stress exposure favors in later onset of irritable bowel syndrome-like symptoms in male mice
Source: Sci Rep. 2021 Aug 11;11:16276. doi: 10.1038/s41598-021-95916-5 (PMC8357959; doi:10.1038/s41598-021-95916-5)
Supplement: Supplementary file 1 — Supplementary Figure. [file 41598_2021_95916_MOESM1_ESM.docx]

**Juvenile social defeat stress exposure favors later onset of irritable bowel syndrome-like symptoms in mice**

Author names

Kenjiro Matsumoto, Kana Takata, Daisuke Yamada, Haruki Usuda, Koichiro Wada, Maaya Tada, Yoshiyuki Mishima, Shunji Ishihara, Syunji Horie, Akiyoshi Saitoh, and Shinichi Kato

Corresponding author

Kenjiro Matsumoto, Division of Pathological Sciences, Department of Pharmacology and Experimental Therapeutics, Kyoto Pharmaceutical University, Misasagi 5, Yamashina, Kyoto 607-8414, Japan.

E-mail: kenjiro@mb.kyoto-phu.ac.jp


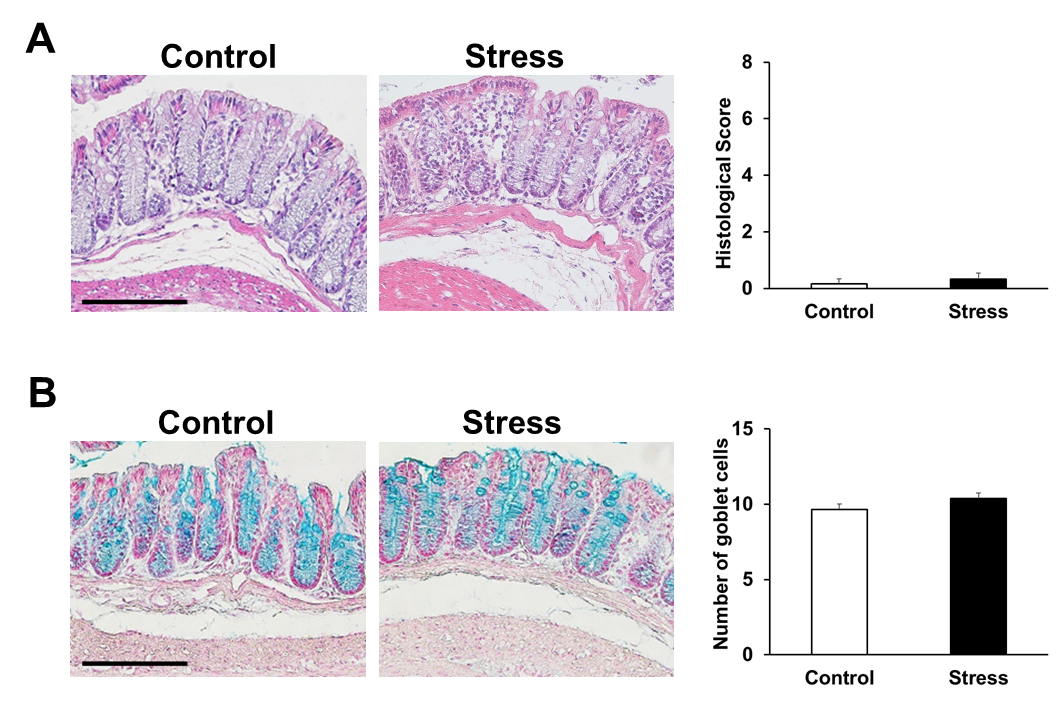


Supplementary Figure 1

Juvenile SDS did not affect histological observation and mucus secretion. Representative images of colonic mucosa with haematoxylin and eosin (A) and alcian blue staining (B) in control and juvenile SDS (stress) mice. Scale bars are 100 μm. The histological scoring and counting alcian Blue-positive goblet cells was performed in normal and juvenile SDS mice. Data are presented as the mean ± SEM from 7 mice per group.
