# Supplementary material for: Juvenile social defeat stress exposure favors in later onset of irritable bowel syndrome-like symptoms in male mice
Source: Sci Rep. 2021 Aug 11;11:16276. doi: 10.1038/s41598-021-95916-5 (PMC8357959; doi:10.1038/s41598-021-95916-5)
Supplement: Supplementary file 2 — Supplementary Tables. [file 41598_2021_95916_MOESM2_ESM.docx]

**Juvenile social defeat stress exposure favors later onset of irritable bowel syndrome-like symptoms in mice**

Author names

Kenjiro Matsumoto, Kana Takata, Daisuke Yamada, Haruki Usuda, Koichiro Wada, Maaya Tada, Yoshiyuki Mishima, Shunji Ishihara, Syunji Horie, Akiyoshi Saitoh, and Shinichi Kato

Corresponding author

Kenjiro Matsumoto, Division of Pathological Sciences, Department of Pharmacology and Experimental Therapeutics, Kyoto Pharmaceutical University, Misasagi 5, Yamashina, Kyoto 607-8414, Japan.

E-mail: kenjiro@mb.kyoto-phu.ac.jp

**Supplementary Table 1** Noradrenaline (NA), 3-methoxy-4-hydroxyphenylglycol (MHPG) content and MHPG/NE ratio in the brain of control and juvenile SDS mice in non-restraint stress (NRS) or restraint stress (RS) condition.

|  | Grouping | FCx | HPC | HYP | AMY | STR | LIM |
| --- | --- | --- | --- | --- | --- | --- | --- |
| NA | Control, NRS | 0.85 ± 0.05 | 0.88 ± 0.20 | 1.52 ± 0.08 | 0.82 ± 0.03 | 0.22 ± 0.07 | 0.95 ± 0.06 |
| (ng/mL) | SDS, NRS | 0.86 ± 0.06 | 0.87 ± 0.15 | 1.36 ± 0.32 | 0.84 ± 0.03 | 0.23 ± 0.07 | 0.96 ± 0.07 |
|  | Control, RS | 0.80 ± 0.05 | 0.68 ± 0.03 | 1.10 ± 0.17 | 0.65 ± 0.02^*^ | 0.21 ± 0.04 | 0.92 ± 0.06 |
|  | SDS, RS | 0.83 ± 0.05 | 0.72 ± 0.05 | 1.30 ± 0.10 | 0.60 ± 0.09^*^ | 0.20 ± 0.06 | 0.91 ± 0.03 |
| MHPG | Control, NRS | 1.58 ± 0.14 | 9.66 ± 2.07 | 1.44 ± 0.39 | 8.06 ± 1.33 | 14.36 ± 2.58 | 1.36 ± 0.10 |
| (ng/mL) | SDS, NRS | 1.41 ± 0.07 | 7.80 ± 1.69 | 1.21 ± 0.48 | 4.95 ± 0.71 | 28.76 ± 11.89 | 1.19 ± 0.10 |
|  | Control, RS | 1.89 ± 0.14 | 7.85 ± 1.24 | 1.71 ± 0.51 | 7.14 ± 1.03 | 26.82 ± 12.68 | 1.79 ± 0.16^*^ |
|  | SDS, RS | 1.82 ± 0.16 | 5.80 ± 0.72 | 1.65 ± 0.32 | 7.53 ± 1.44^*^ | 15.44 ± 2.20 | 1.60 ± 0.06^*^ |
| MHPG/NA | Control, NRS | 1.84 ± 0.11 | 11.96 ± 3.09 | 0.90 ± 0.19 | 10.11 ± 1.94 | 69.71 ± 13.55 | 1.44 ± 0.08 |
|  | SDS, NRS | 1.68 ± 0.10 | 10.63 ± 3.16 | 0.67 ± 0.26 | 5.86 ± 0.84 | 152.79 ± 53.70 | 1.28 ± 0.14 |
|  | Control, RS | 2.36 ± 0.16^*^ | 11.80 ± 2.42 | 1.37 ± 0.39 | 11.10 ± 1.81 | 129.92 ± 49.18 | 1.96 ± 0.15^*^ |
|  | SDS, RS | 2.17 ± 0.07^*^ | 7.94 ± 0.67 | 1.30 ± 0.25 | 12.93 ± 1.94^*^ | 83.61 ± 5.37 | 1.76 ± 0.06^*^ |

FCx, frontal cortex; HPC, hippocampus; HYP, hypothalamus; AMY, amygdala; STR, striatum; LIM, limbic area. Data are presented as the mean ± SEM for 8 mice per group. *P < 0.05 for comparison with the no restrained group.

**Supplementary Table 2** Dopamine (DA), homovanillic acid (HVA), 3,4-dihydroxyphenylacetic acid (DOPAC) content, and (DOPAC+HVA)/DA ratio in the brain of control and juvenile SDS mice in non-restraint stress (NRS) or restraint stress (RS) condition.

|  | Grouping | FCx | HPC | HYP | AMY | STR | LIM |
| --- | --- | --- | --- | --- | --- | --- | --- |
| DA | Control, NRS | 1.37 ± 0.25 | 0.01 ± 0.01 | 0.39 ± 0.04 | 0.90 ± 0.10 | 15.7 ± 2.2 | 10.04 ± 0.29 |
| (ng/mL) | SDS, NRS | 0.97 ± 0.16 | 0.05 ± 0.04 | 0.36 ± 0.08 | 0.87 ± 0.11 | 9.2 ± 2.2 | 10.58 ± 0.68 |
|  | Control, RS | 1.56 ± 0.36 | 0.02 ± 0.01 | 0.43 ± 0.07 | 0.91 ± 0.11 | 14.0 ± 3.3 | 10.36 ± 0.24 |
|  | SDS, RS | 1.33 ± 0.25 | 0.03 ± 0.00 | 0.48 ± 0.04 | 0.94 ± 0.19 | 13.0 ± 2.7 | 10.97 ± 0.32 |
| DOPAC | Control, NRS | 0.29 ± 0.04 | 0.53 ± 0.16 | 0.21 ± 0.02 | 0.07 ± 0.01 | 0.93 ± 0.13 | 0.76 ± 0.05 |
| (ng/mL) | SDS, NRS | 0.27 ± 0.02 | 0.33 ± 0.08 | 0.18 ± 0.05 | 0.14 ± 0.05 | 0.89 ± 0.15 | 0.84 ± 0.09 |
|  | Control, RS | 0.37 ± 0.07 | 0.31 ± 0.05 | 0.25 ± 0.05 | 0.22 ± 0.04^*^ | 0.81 ± 0.21 | 1.27 ± 0.07^*^ |
|  | SDS, RS | 0.34 ± 0.04 | 0.27 ± 0.05 | 0.29 ± 0.03 | 0.19 ± 0.06 | 0.82 ± 0.17 | 1.33 ± 0.13^*^ |
| HVA | Control,NRS | 0.58 ± 0.07 | 0.66 ± 0.16 | 0.23 ± 0.03 | 0.37 ± 0.03 | 2.69 ± 0.33 | 1.19 ± 0.04 |
| (ng/mL) | SDS, NRS | 0.54 ± 0.05 | 0.89 ± 0.33 | 0.19 ± 0.04 | 0.35 ± 0.05 | 4.64 ± 1.57 | 1.16 ± 0.07 |
|  | Control, RS | 0.65 ± 0.05 | 0.55 ± 0.18 | 0.30 ± 0.05^*^ | 0.54 ± 0.04^*^ | 3.72 ± 0.47 | 1.83 ± 0.03^*^ |
|  | SDS, RS | 0.63 ± 0.06 | 0.24 ± 0.06 | 0.33 ± 0.02^*^ | 0.52 ± 0.09^*^ | 3.63 ± 0.44 | 1.71 ± 0.03^*^ |
| (DOPAC+HVA)/DA | Control, NRS | 0.69 ± 0.06 | 241.89 ± 101.78 | 1.13 ± 0.07 | 0.53 ± 0.08 | 0.28 ± 0.06 | 0.19 ± 0.01 |
|  | SDS, NRS | 1.14 ± 0.35 | 1323.36 ± 959.87 | 0.79 ± 0.18 | 0.56 ± 0.06 | 2.25 ± 1.52 | 0.19 ± 0.01 |
|  | Control, RS | 0.93 ± 0.21 | 258.71 ± 232.71 | 1.13 ± 0.18 | 0.86 ± 0.05^*^ | 0.56 ± 0.17 | 0.30 ± 0.01^*^ |
|  | SDS, RS | 0.91 ± 0.18 | 22.34 ± 9.56 | 1.36 ± 0.13 | 0.83 ± 0.11^*^ | 0.45 ± 0.10 | 0.28 ± 0.02^*^ |

FCx, frontal cortex; HPC, hippocampus; HYP, hypothalamus; AMY, amygdala; STR, striatum; LIM, limbic area. Data are presented as the mean ± SEM for 8 mice per group. *P < 0.05 for comparison with the no restrained group.

**Supplementary Table 3** Serotonin (5-HT), 5-hydroxyindole acetic acid (5-HIAA) content, and 5-HIAA/5-HT ratio in the brain of control and juvenile SDS mice in non-restraint stress (NRS) or restraint stress (RS) condition.

|  | grouping | FCx | HPC | HYP | AMY | STR | LIM |
| --- | --- | --- | --- | --- | --- | --- | --- |
| 5-HT | Control, NRS | 0.96 ± 0.15 | 1.54 ± 0.32 | 1.02 ± 0.32 | 3.14 ± 0.86 | 0.06 ± 0.06 | 0.71 ± 0.20 |
| (ng/mL) | SDS, NRS | 1.10 ± 0.08 | 1.71 ± 0.23 | 1.17 ± 0.33 | 2.08 ± 0.76 | - | 0.61 ± 0.20 |
|  | Control, RS | 0.99 ± 0.15 | 1.79 ± 0.19 | 0.78 ± 0.20 | 1.92 ± 0.68 | 0.18 ± 0.12 | 1.02 ± 0.19 |
|  | SDS, RS | 1.05 ± 0.14 | 1.72 ± 0.47 | 1.14 ± 0.18 | 1.68 ± 0.63 | - | 0.67 ± 0.21 |
| 5-HIAA | Control, NRS | 0.25 ± 0.04 | 1.68 ± 0.19 | 0.47 ± 0.13 | 1.56 ± 0.35 | 8.54 ± 1.46 | 0.22 ± 0.05 |
| (ng/mL) | SDS, NRS | 0.25 ± 0.02 | 1.74 ± 0.26 | 0.54 ± 0.18 | 1.19 ± 0.39 | 5.14 ± 1.32 | 0.18 ± 0.05 |
|  | Control, RS | 0.40 ± 0.07^*^ | 2.51 ± 0.17 | 0.48 ± 0.10 | 1.70 ± 0.49 | 5.53 ± 1.03 | 0.59 ± 0.09^*^ |
|  | SDS, RS | 0.43 ± 0.06^*^ | 2.24 ± 0.66 | 0.63 ± 0.08 | 1.35 ± 0.44 | 8.58 ± 1.23 | 0.36 ± 0.09 |
| 5-HIAA/5-HT | Control, NRS | 0.26 ± 0.01 | 1.22 ± 0.20 | 0.52 ± 0.06 | 0.72 ± 0.13 | - | 0.35 ± 0.04 |
|  | SDS, NRS | 0.23 ± 0.01 | 1.03 ± 0.10 | 0.34 ± 0.08 | 0.74 ± 0.10 | - | 0.29 ± 0.05 |
|  | Control, RS | 0.38 ± 0.03^*^ | 1.43 ± 0.09 | 0.52 ± 0.13 | 1.24 ± 0.19 | - | 0.62 ± 0.03^*^ |
|  | SDS, RS | 0.41 ± 0.01^*^ | 1.27 ± 0.12 | 0.60 ± 0.07 | 1.35 ± 0.43 | - | 0.67 ± 0.09^*^ |

FCx, frontal cortex; HPC, hippocampus; HYP, hypothalamus; AMY, amygdala; STR, striatum; LIM, limbic area. Data are presented as the mean ± SEM for 8 mice per group. *P < 0.05 for comparison with the no restrained group.
